# Evaluating Brain Tumor Detection with Deep Learning Convolutional Neural Networks Across Multiple MRI Modalities

**DOI:** 10.3390/jimaging10120296

**Published:** 2024-11-21

**Authors:** Ioannis Stathopoulos, Luigi Serio, Efstratios Karavasilis, Maria Anthi Kouri, Georgios Velonakis, Nikolaos Kelekis, Efstathios Efstathopoulos

**Affiliations:** 1 2nd Department of Radiology, Medical School, Attikon University Hospital, National and Kapodistrian University of Athens, 11527 Athens, Greece; ianstath@med.uoa.gr (I.S.); mariakouri90@gmail.com (M.A.K.); gvelonakis@med.uoa.gr (G.V.); kelnik@med.uoa.gr (N.K.); 2 Technology Department, CERN, 1211 Geneva, Switzerland; luigi.serio@cern.ch; 3 Medical Physics Laboratory, School of Medicine, Democritus University of Thrace, 68100 Alexandroupolis, Greece; stratoskaravasilis@yahoo.gr

**Keywords:** brain tumors, central nervous system (CNS) tumors, MRI, transfer learning, convolutional neural networks (CNNs)

## Abstract

Central Nervous System (CNS) tumors represent a significant public health concern due to their high morbidity and mortality rates. Magnetic Resonance Imaging (MRI) has emerged as a critical non-invasive modality for the detection, diagnosis, and management of brain tumors, offering high-resolution visualization of anatomical structures. Recent advancements in deep learning, particularly convolutional neural networks (CNNs), have shown potential in augmenting MRI-based diagnostic accuracy for brain tumor detection. In this study, we evaluate the diagnostic performance of six fundamental MRI sequences in detecting tumor-involved brain slices using four distinct CNN architectures enhanced with transfer learning techniques. Our dataset comprises 1646 MRI slices from the examinations of 62 patients, encompassing both tumor-bearing and normal findings. With our approach, we achieved a classification accuracy of 98.6%, underscoring the high potential of CNN-based models in this context. Additionally, we assessed the performance of each MRI sequence across the different CNN models, identifying optimal combinations of MRI modalities and neural networks to meet radiologists’ screening requirements effectively. This study offers critical insights into the integration of deep learning with MRI for brain tumor detection, with implications for improving diagnostic workflows in clinical settings.

## 1. Introduction

A brain tumor, or brain cancer, is an abnormal and uncontrolled proliferation of brain cells, often associated with high morbidity and mortality rates. According to the World Health Organization (WHO), brain cancer accounts for approximately 2% of all human cancers [1,2]. Thus, early and accurate diagnosis of brain lesions is essential for selecting optimal treatment strategies, which can either directly treat the condition, prolong survival, or improve quality of life [3]. Magnetic Resonance Imaging (MRI) has become central to the accurate diagnosis of various brain abnormalities [4]. As a non-invasive and radiation-free imaging modality, MRI enables the detection and characterization of brain tissue abnormalities by differentiating tissues on the basis of their distinct magnetic properties, visualized through various grayscale contrasts depending on the imaging technique used [5]. However, the sensitivity and specificity of different MRI techniques vary across brain lesions.

**Figure 1:**
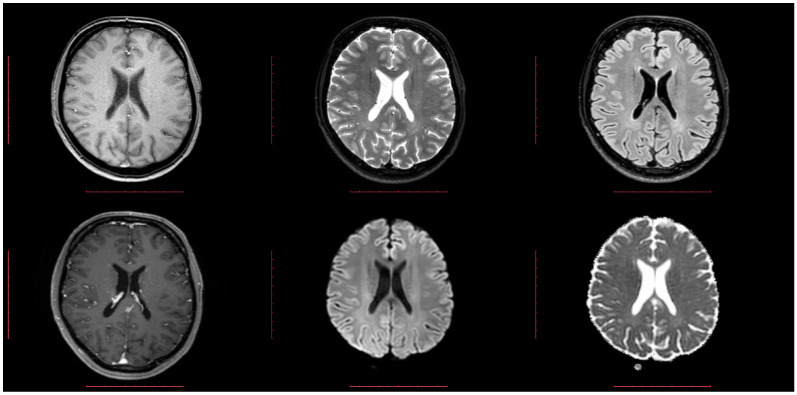
Six different MRI sequences of a normal brain examination. From left to right and top to bottom: T1, T2, FLAIR, T1+C, Diffusion, apparent diffusion coefficient (ADC) map.

**Figure 2:**
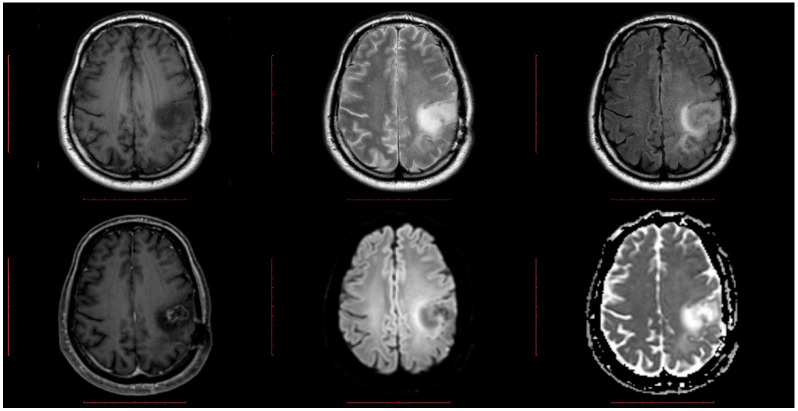
Six different MRI sequences of a verified Brain Tumor examination. From left to right and top to bottom: T1, T2, FLAIR, T1+C, Diffusion, and ADC.

Standard clinical brain MRI protocols leverage multiple sequences, each serving unique diagnostic purposes due to their sensitivity to different tissue characteristics. T2-weighted (T2) images are commonly used to highlight areas of high water content. FLAIR (T2-weighted with fluid-attenuated inversion recovery) further enhances this by suppressing signals from cerebrospinal fluid, which improves the visibility of periventricular lesions. Diffusion-weighted imaging (DWI) and the apparent diffusion coefficient (ADC) map are essential for assessing the movement of water molecules within tissues by highlighting regions of restricted diffusion. T1-weighted (T1) sequences provide clear anatomical details and are frequently combined with gadolinium contrast (T1+C) to identify disruptions in the blood-brain barrier, which is valuable for detecting tumors, inflammation, and areas of active disease (Figure 1 and Figure 2) [6]. Additionally, advanced MRI techniques, such as perfusion imaging, magnetic resonance spectroscopy, diffusion tensor imaging, and amide proton transfer (APT) weighted imaging, are incorporated into oncological brain imaging to improve diagnostic accuracy in tissue characterization [7]. Each sequence contributes distinct information, and together, they offer a comprehensive view of brain pathology by capitalizing on the varied signal properties across tissue types and disease states.

Inevitably, the increasing demand for MRI scans, particularly for brain evaluation, has led to a substantial workload for radiologists, who must often analyze and interpret numerous images manually. As a result, radiologists are required to visually examine thousands of images daily, which can make the diagnostic process both time-consuming and prone to error. Manual assessment can introduce subjectivity, which may impact diagnostic consistency and accuracy. However, while manual interpretation carries the risk of human error, CNN-based computer-aided diagnosis systems also introduce their own sources of error, primarily due to model limitations, biases in training data, and potential misclassifications. Compared to manual assessments, errors from CNNs tend to be systematic, often reflecting the model’s exposure to specific data patterns during training. In this context, when radiologists understand and mitigate their error patterns, computer-aided diagnosis systems could offer an opportunity to assist them, streamline reporting times, and minimize the risk of diagnostic errors [8,9].

Over recent years, machine learning and deep learning approaches have demonstrated significant promise in the automated detection of brain pathology [9,10,11,12,13,14]. For instance, Amarnath et al. [15] achieved nearly 98% classification accuracy on brain MRI images using transfer learning with the Xception model, without particular MRI sequence discrimination. Similarly, Muhammed Talo et al. [16] reported 100% accuracy using a pre-trained ResNet34 model on T2-weighted MRIs. In both studies, the MRI sequences were considered particularly suitable for lesion detection, though the datasets were not tumor-exclusive, lacked extensive model comparisons, and did not incorporate the diagnostic value of multiple MRI sequences. This underscores the need for a broader, comparative approach to evaluate MRI sequence effectiveness across diverse model options.

In this study, we investigate and benchmark six commonly used MRI sequences in conjunction with four deep learning convolutional neural networks (CNNs) to evaluate the clinical value and diagnostic impact of each sequence in developing an automated screening system. This system aims to accurately classify 2D MRI slices as either “normal” or “tumorous”, which could support more efficient and accurate radiological workflows.

To the best of our knowledge, this is among the first studies to comprehensively assess the precision, accuracy, specificity, and sensitivity of six distinct MRI sequences through various deep learning models and transfer learning methods using a balanced, tumor-specific dataset. This approach hopes to provide a focused evaluation of different MRI modalities within a dedicated tumor imaging context, and thus offering insights beyond those in studies involving broader or mixed-pathology datasets. Our findings aim to delve deeper into the future development of reliable, automated screening tools with the potential to reduce diagnostic workloads and enhance clinical accuracy.

## 2. Materials and Methods

### 2.1. Dataset Collection and Preparation

Sixty-two brain MRI studies with tumor findings were collected. Brain MRIs were performed on a 3.0T Achieva TX Philips MRI system at Aiginiteio University Hospital, Athens. The MRI system is equipped with an eight-channel head coil, while the brain imaging protocol includes both conventional and advanced imaging techniques. T2 weighted turbo spin echo and gradient echo sequences, T2 FLAIR, DWI, T1 weighted pre- and post-contrast, T2* dynamic susceptibility imaging, diffusion tensor imaging, and single-voxel and 2D Magnetic resonance spectroscopy sequences were applied in all the participants. Experienced MR physicists visually inspected images for the presence of potential artifacts, and experienced neuroradiologists evaluated and reported the studies. As mentioned, from the above imaging data, we analyzed the T2, FLAIR, T1, T1+C, native DWI (Diffusion), and diffusion-derived Apparent Coefficient map (ADC).

The dataset was divided into a training set of 52 patients and a testing set of 10 patients. From each MRI sequence, multiple 2D axial slices containing both tumor and normal tissue were extracted. The final distribution was achieved by excluding slices containing artifacts and other pathologies that were seriously deformed due to surgeries or any other type of therapy. Table 1 provides a detailed overview of the training and testing sets per sequence. In total, seven subsets were created: one subset for each MRI sequence analyzed and an additional subset containing the complete set of images for each class.

### 2.2. Image Preprocessing

Image preprocessing and data augmentation are essential for effectively utilizing CNNs in the medical field, as they significantly enhance performance, reduce data dimensionality, lower computational complexity, and shorten processing time [17]. The MRI images in the dataset underwent the following preprocessing steps: the 2D-pixel arrays of the DICOM slices were retained, and most black pixels surrounding the brain were cropped, aligning the skull’s borders with the image edges. All images were then resized to dimensions of 224 × 224 × 1.

Normalization was performed on the images using the following equation:

(1) $$p_{normalized}=\frac{p-\mu }{\sigma }$$

where the following is defined:

- *p* is the original pixel intensity value;
- *μ* is the mean of all pixel values in the image;
- *σ* is the standard deviation of all pixel values in the image.

**Figure 3:**
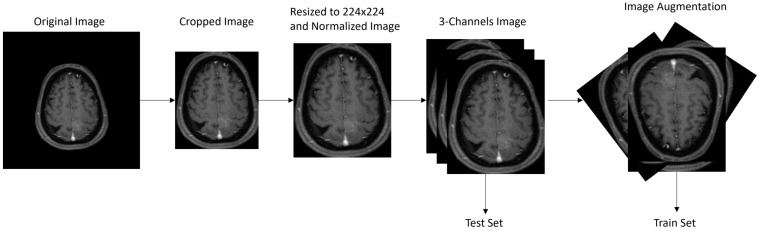
Image representation of the preprocessing steps.

Training images were augmented through horizontal and vertical flips, rotations within a ±90° range, random zooming from 0% to 20%, and shuffling, presenting them to the models as new samples. In contrast, the test sets were neither augmented nor shuffled. Since the MRI images were grayscale and our models required three-channel input, the grayscale values were replicated three times to achieve dimensions of 224 × 224 × 3 (Figure 3).

### 2.3. CNN Models—Transfer Learning

Four distinct CNN models were trained and tested for the classification task of differentiating between normal and tumor slices: VGG16 [18], MobileNetV2 [19], ResNet50 [20], and InceptionV3 [21]. Each of these models has made significant contributions to the deep learning field since their introduction, achieving state-of-the-art performance across various computer vision tasks. They utilize characteristics of CNNs’ structures for MRI deep learning applications [22], as can be depicted in Table 2.

Training and tuning the above models from scratch could prove to be time and computing power-consuming, especially with small datasets such as the ones of the medical domain. Transfer learning was employed to solve this problem [23,24,25,26,27,28]. We initiated all four models with pre-trained weights from the Large-Scale Visual Recognition Challenge (ILSVRC), which uses a subset of ImageNet of almost 1.2 million natural training images [29]. Furthermore, for our classification task, we removed the top-classification layer from all four models, and after a trial-and-error study for the parameter’s selection, we added a home-build classifier, which sequentially consists of the following:

1. A global average pooling layer;
2. First Dense Layer with 96 neurons, RELU activation function and kernel regularizes l1 = 0.3 and l2 = 0.3;
3. Dropout Layer with 40% random neuron rejection;
4. Batch normalization Layer;
5. Second Dense Layer with 96 neurons, RELU activation functions, and kernel regularizes l1 = 0.5 and l2 = 0.4;
6. Dropout Layer with 40% random neuron rejection;
7. Batch normalization Layer;
8. SoftMax Dense Layer with 2 classes of output.

All four CNNs were left to train under the hyper-parameters presented in Table 3. For the training, testing, and overall implementation of the models, we used TensorFlow with Keras API [30] and an NVIDIA GPU Quadro K2200 (NVIDIA Corporation, Santa Clara, California, USA) with 4 GB RAM, along with an Intel Xeon E5-1630v3 @3.70Hz (Intel Corporation, Santa Clara, California, USA) with 32GB RAM and Windows 11 (Microsoft Corporation, Redmond, Washington, USA) operating system.

### 2.4. Model’s Evaluation Metrics and Methods

We used various evaluation metrics to assess the results as described below:

- Precision = TP/(TP + TN)%;
- Sensitivity = TP/(TP + FN)%;
- Specificity = TN/(TN + FN)%;
- Accuracy = (TP + TN)/(TP + TN + FP + FN)%.

True Positive (TP) is the number of predicted tumorous images that are tumorous. True Negative (TN) is the number of normal predicted images, and they are normal. False Negative (FN) is the number of normal predicted cases while they are tumorous, and False Positive (FP) is the number of tumorous predicted cases while they are normal. Finally, we used the ROC curve and the AUC score as evaluation metrics for checking and comparing the different classification model’s performance across the different MRI sequences.

## 3. Results

Seven different runs were performed per model: one for each sequence and one with the total amount of images regardless of sequence. All classification metrics were measured over the 199 tumorous and 123 normal images of the test set.

The results for accuracy ranged from 80 to 98.4% across the models depending on the sequence. VGG16 presented accuracy levels of more than 96% at FLAIR and T1+C, MobilenetV2 of more than 95% at ADC, and InceptionV3 at almost 94% at T1. On average, the Resnet50 model had the most robust results across all sequences, and the Flair sequence had the best accuracy performance across all models, as can be depicted in Table 4.

In the sensitivity analysis, T1 was the best sequence for detecting the tumorous slices with a 100% ratio across all models. Meanwhile, all other sequences, apart from T2, had, on average, sensitivity results above 93%. On average, the most sensitive models were VGG16 and Resnet50 (Table 5).

On the tradeoff metric of specificity and the ability to correctly detect the normal slices, it was characteristic that only FLAIR and T1+C sequences performed above 90%. There was a specificity drop for FLAIR, ADC, T1, and Diffusion, while only T2 and -marginally- T1+C had better specificity than sensitivity performance. MobileNetV2 was the model with the best average specificity across all sequences (Table 6).

Concerning the precision results, FLAIR and T1+C, once again, showed a performance of around 95% across all models, with VGG16 having 100% precision at both sequences (Table 7).

Lastly, the results of the AUC scores are presented in Table 8. We noticed strong performance at FLAIR and T1+C for all models, especially for VGG16, which gave AUCs of 1 and 0.99, respectively. MobilenetV2 reached 0.98 and 0.92 at ADC and T1, respectively, while Resnet50 reached around 0.90 at Diffusion and T2.

Following the AUC scores, the ROC curves can provide valuable and complementary information on performance at different true positive and false positive rates for each model and each sequence.

**Scheme 1:**
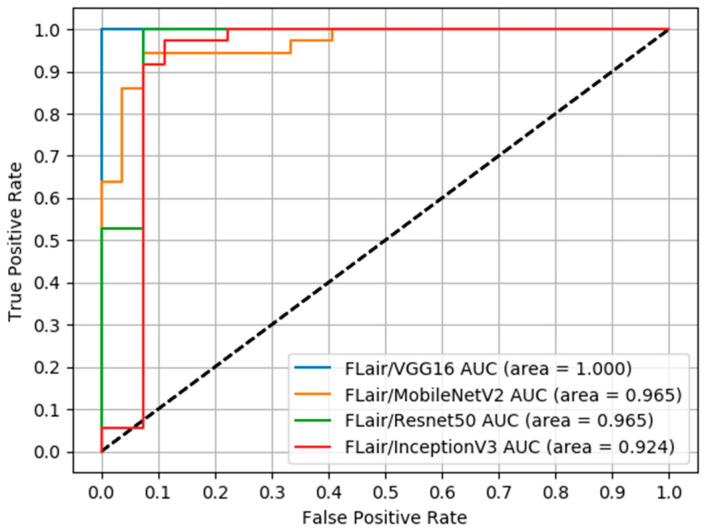
ROCs for FLAIR sequence.

**Scheme 2:**
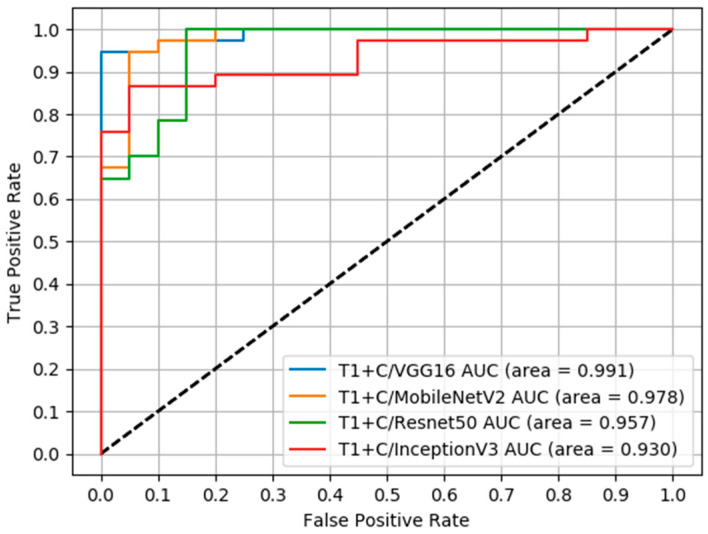
ROCs for T1+C sequence.

The ROCs for FLAIR presented a robust behavior for all models; for false positive rates (FPR) of 0–8%, all models returned 92–100% true positive rates (TPR) (Scheme 1). Similarly, at T1+C, we noticed 88–100% TPRs and 0–15% FPRs (Scheme 2).

**Scheme 3:**
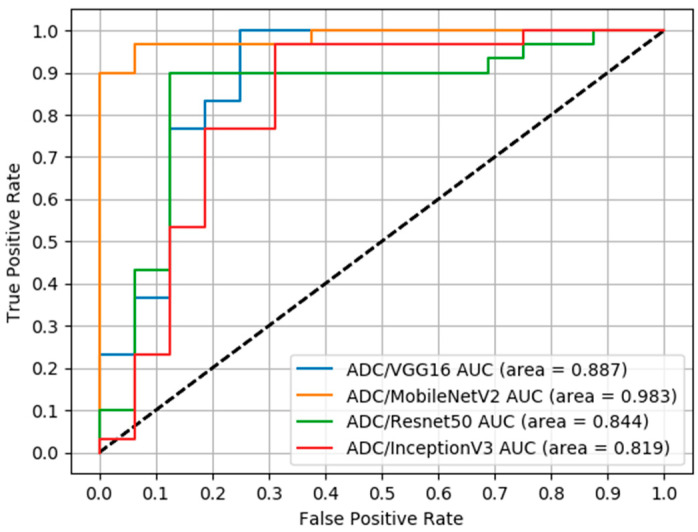
ROCs for ADC sequence.

**Scheme 4:**
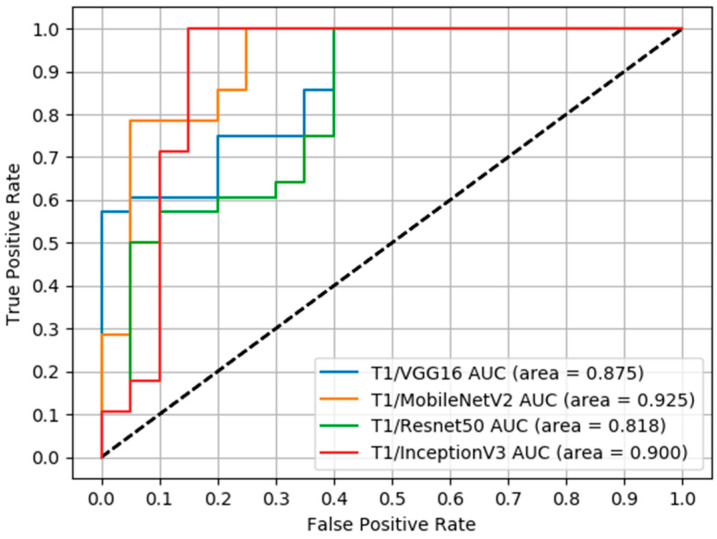
ROCs for T1 sequence.

The ROCs for the ADC sequence gave TPRs of 78–98% at 20% FPR. The MobileNetV2 model was an exception, with a 98% TPR at 7% FPR (Scheme 3). At the T1 sequence, the InceptionV3 stood out, reaching a TPR of 100% at 15% FPR (Scheme 4).

**Scheme 5:**
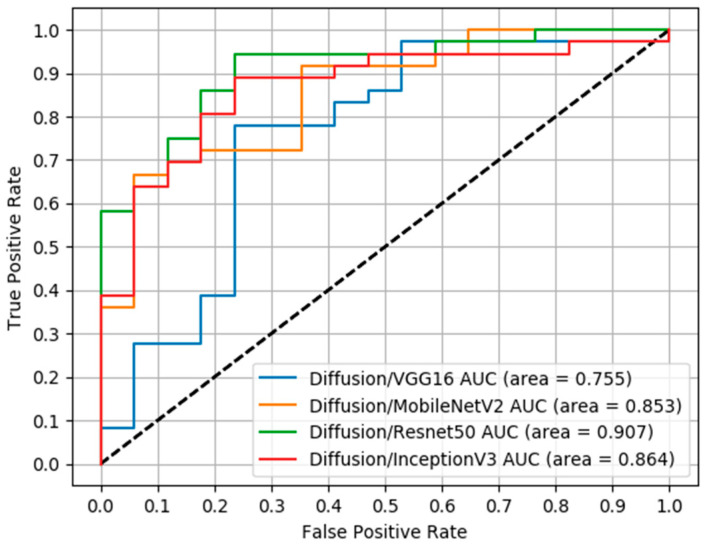
ROCs for Diffusion sequence.

**Scheme 6:**
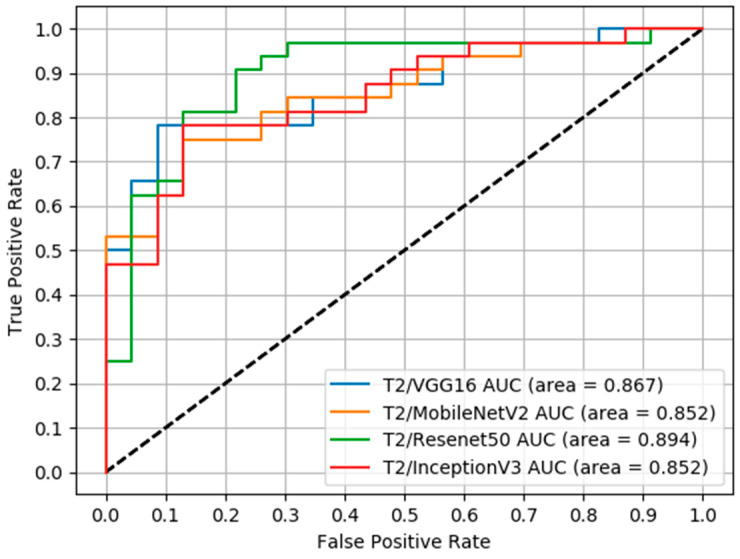
ROCs for T2 sequence.

For Diffusion and T2 sequences, the ROCs followed similar results and distributions, with TPRs of 65–85% and FPRs of 10–20% (Scheme 5 and Scheme 6).

**Scheme 7:**
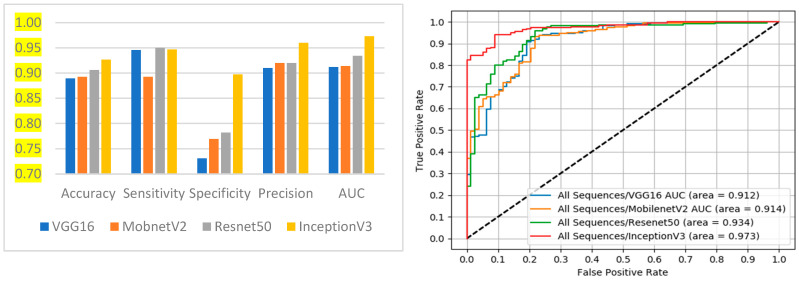
(**Left**): The evaluation metrics results of the experiment are in the whole dataset. (**Right**): the corresponding ROC curve.

In the final experiment, the models were trained and tested on datasets that combined all the sequences together. This approach aimed to examine the models’ performance on a more complex dataset, exposing them to all MRI sequences simultaneously during the training phase. There, Resnet50 and InceptionV3 performed better, showing an accuracy of 91% and 93%, respectively, and AUC scores above 0.93 and 0.97, respectively (Scheme 7).

## 4. Discussion

Magnetic Resonance Imaging (MRI) plays a vital role in the diagnosis of brain tumors, and optimizing imaging techniques is essential for enhancing diagnostic precision. In this study, we aimed to leverage the strengths of six widely used clinical MRI sequences to develop a deep learning tool for the automated classification of tumorous and normal MRI slices. Our focus was on identifying the optimal model among four well-established CNN architectures while integrating transfer learning to improve performance and accuracy in clinical applications.

From a clinical point of view, the contrast and the different intensity values between brain regions of interest consist of the main decision-making factors to discriminate a brain lesion from a normal brain area.

The final radiologist’s diagnosis arises from gathering and combining data from all the available image sequences, but some of them are indispensable. FLAIR sequence provides inherently increased contrast due to the suppression of the fluid signals highlighting the lesion and its possible edema. Increased visibility of lesions is also achieved in post-contrast T1 weighted sequences in the case of gadolinium-enhancing lesions. However, many neoplasms, especially (but not only) low-grade tumors, do not enhance after contrast administration; thus, their visibility is not increased on T1 post-contrast imaging. We assume that this is a limitation of the studies using exclusively T1 post-contrast images. However, these two sequences are needed for both lesion detection and characterization. T1 pre-contrast and T2 sequences are also useful for lesion evaluation and estimation of potential peripheral edema (with lower contrast compared to the FLAIR sequence) and depiction of intratumoral hemorrhage. Similarly, the advanced sequences of Diffusion and ADC are mainly used by radiologists for differentiation among tumor types and grades and not for normal and tumorous slice classification [31,32,33,34].

### 4.1. Sequence-Wise Analysis

The accuracy order of the sequences, with respect to our results, follows the clinical pattern and shows a direct relationship between the deep learning analysis and clinical use.

According to the literature, FLAIR and T1+C are among the most common MRI sequences used for similar deep learning applications, giving very reliable results [35,36,37]. Our results confirm the above as well. FLAIR and T1+C showed remarkably robust performance compared to the other sequences with results at all metrics and models, particularly with VGG16, which can directly support the clinical purpose of distinction between normal and tumorous MRI images and fulfill the clinical need for accurate tumor detection and characterization.

However, the usage of the remaining imaging sequences can potentially bring complementary clinical values. For tumor screening applications with high sensitivity requirements and edema or hemorrhage information needs, the T1 sequence with the InceptionV3 model or T2 with VGG16 are the best options. For high accuracy and tumor differentiation options, the combination of ADC with MobileNetV2 or Diffusion with Resnet50 presents promising results.

Additionally, the ROC analysis shows the flexibility and robustness of the different models across sequences, offering the end-user radiologist the option to decide the optimal value of FPR according to his TPR needs.

### 4.2. Model-Wise Analysis

In the modeling analysis, VGG16, despite achieving high accuracy, faces limitations in specificity, particularly in sequences like ADC, T1, and Diffusion, where its simpler architecture may struggle to capture nuanced differences in non-tumorous tissues. This highlights a potential tradeoff for VGG16; its straightforward structure and interpretability provide strong accuracy on some sequences but may reduce specificity in cases requiring subtle differentiations. In contrast, MobileNetV2, Resnet50, and InceptionV3 demonstrate more consistent and robust performance across complex sequences, with smaller standard deviations in both sensitivity and specificity. These models, which have deeper architectures, likely benefit from their capacity to capture finer details and more complex patterns, resulting in stable performance across diverse sequence types.

Additionally, the final experiment, which combined all sequences into a single training set, underscores the capability of deep CNNs, particularly InceptionV3, to achieve reliable diagnostic performance on multisequence datasets without needing initial sequence separation. InceptionV3’s ability to reach an AUC above 0.97 in this combined setting (Scheme 7) suggests that such deep networks could be valuable in time-sensitive clinical situations. This combined sequence approach makes it possible to screen multiple MRI types in a single pass, which could be highly beneficial in clinical emergencies when immediate assessment across sequences is necessary, but time for extensive preprocessing is limited.

### 4.3. Explainability and Clinical Correlation

**Figure 4:**
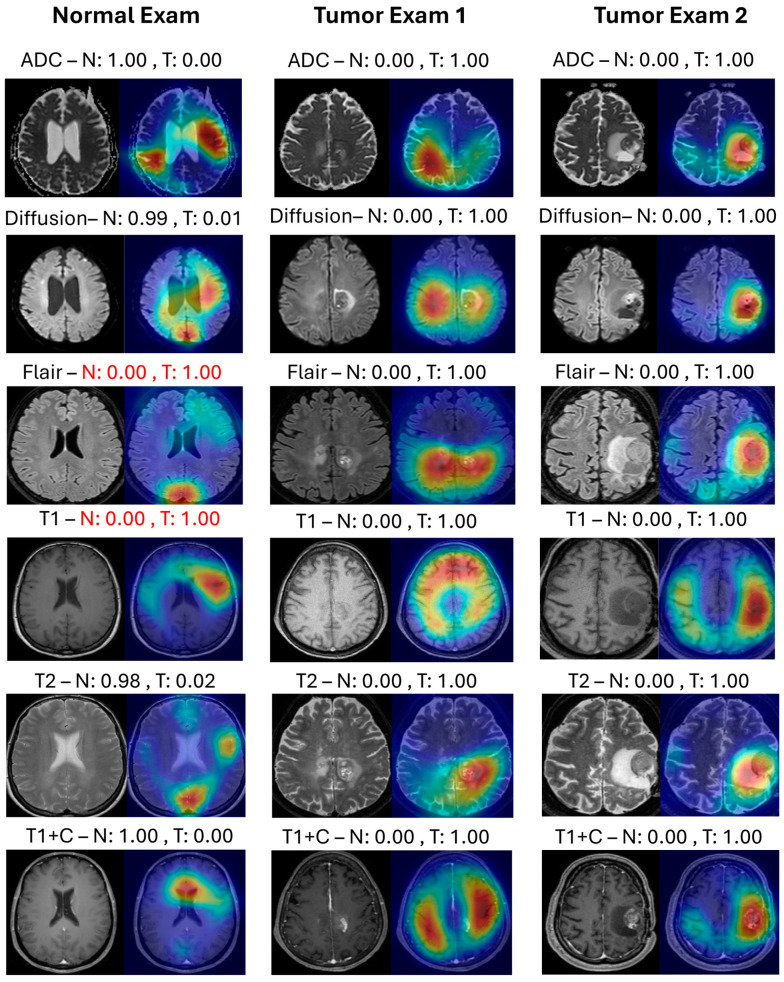
One normal and two tumor examinations are shown for all six MRI sequences. In all images, the original image is displayed on the left, and the overlap with the heatmap produced from the last convolutional layer of the VGG16 model is displayed on the right. In the titles, N represents the Normal class, and T represents the Tumor class, both followed by the prediction probability for the respective class. Misclassified cases are highlighted in red.

During diagnostic procedures, clinicians and radiologists examine all available MRI sequences, both individually for specific anatomical regions and comparatively across sequences, to reach a final diagnosis. Occasionally, abnormalities may appear in some sequences but not in others. To explore whether CNN models follow similar reasoning and to emphasize the importance of including as many sequences as possible in the training set, we generated heatmaps [38] and prediction probabilities for the same 2D axial slice across one normal and two tumor examinations for all six MRI sequences (Figure 4). We utilized the VGG16 model trained on all sequences, as it demonstrated the highest accuracy and has a relatively simple architecture.

For the normal examination, the model misclassified two slices (FLAIR and T1) as tumorous but correctly classified the other four. By applying a majority vote, the examination could still be accurately identified as normal, mimicking the interpretative process of human experts. Additionally, the heatmaps for the normal case revealed that the model focused on different anatomical regions in each sequence, showcasing the variety of details each sequence provides.

For the two tumor examinations, the model classified all slices correctly. The heatmaps showed that the model’s focus extended beyond the tumor core to surrounding regions (e.g., ADC and T2 sequences) and even to areas farther away (e.g., T1 and T1+C sequences). This behavior could support radiologists by prompting them to examine areas near the tumor core for micro-infiltrations or tumor expansion and to investigate more distant regions for potential changes in white and gray matter patterns indicative of metastasis.

## 5. Limitations-Future Work

Transferring learning utilization from large-scale annotated natural image datasets (ImageNet) to medical domain problems has been consistently beneficial in coping with a limited amount of data. However, we are focusing on expanding and further balancing the existing datasets by adding more training images, including ones from different MRI systems and medical centers.

The models’ hyper-parameters and the top classification layers were consciously kept identical across the different models for comparison reasons, but there is a need for a future benchmarking of each model separately to further enhance each’s accuracy and performance. Additionally, more complex and advanced base models could improve classification accuracy, with examples being CNNs like Efficientnet [39], transformer-based image classification networks like Vision Transformers [40], and contemporary CNNs like ConvNeXt [41].

Finally, in this study, we experimented using only the clinical case of classification between normal and tumorous slices. Experiments with further clinical cases (i.e., classification of different brain pathologies or tumor differentiation and characterization) could provide more advanced diagnostic tools.

## 6. Conclusions

Numerous studies have demonstrated that deep learning techniques can significantly enhance medical and radiology practices. In our investigation, we examined the influence of six key MRI sequences on the development of an effective tumor screening system. By employing four deep transfer-learning models, we achieved high levels of accuracy, demonstrating that different MRI sequences can support diverse clinical decisions. We propose that a comprehensive deep learning platform equipped with optimal input combinations and model selections has the potential to offer versatile and precise assistance throughout the diagnostic process.

## Figures and Tables

**Table 1:** Overview of the training and testing sets categorized by MRI sequence. A total of seven subsets were generated, including one for each analyzed MRI sequence and an additional subset comprising the complete set of images for each class, distinguishing between tumor-containing and normal samples.

52 Patients Train 10 Patients Test		Class				
		Tumour		Normal		Total
		Train	Test	Train	Test	
Sequences	ADC	146	30	49	16	241
	Diffusion	142	36	63	17	258
	FLAIR	225	36	107	27	395
	T1	78	28	62	20	188
	T1+C	131	37	100	20	288
	T2	155	32	66	23	276
	Total	877	199	447	123	1646

**Table 2:** Overview of the CNN models utilized in this study for classifying normal and tumor MRI slices. Each model has significantly impacted deep learning and exhibits state-of-the-art performance in MRI applications [22].

Model	Network Depth	Size (Mb)	Parameters	Characteristic Structure	Top-1 % Error at ImageNet
VGG16	23	528	138,357,544	Stacked Convolution Blocks	0.713
MobilenetV2	88	14	3,538,984	Inverted Residuals and Linear Bottlenecks	0.713
Resnet50	50	98	25,636,712	Residual Layers	0.749
InceptionV3	159	92	23,851,784	Concatenated Different-Sized Convolutional Filters	0.779

**Table 3:** Summary of hyperparameters used for training the CNN models.

Image Size	224 × 224 × 3
Training Epochs	600
Batch Size	16
Loss Function	Categorical Cross-Entropy
Optimizer	Adam
Learning Rate	0.00001
Choosing Criteria	The model with the best test accuracy

**Table 4:** Accuracy performance of the CNN models across different MRI sequences.

Accuracy	FLAIR	T1+C	ADC	T1	Diffusion	T2	Avg/Sequence	SD
VGG16	0.984	0.965	0.891	0.833	0.811	0.836	0.887	0.073
MobnetV2	0.921	0.930	0.957	0.896	0.830	0.800	0.889	0.061
Resnet50	0.968	0.947	0.891	0.833	0.887	0.836	0.894	0.056
InceptionV3	0.937	0.895	0.870	0.938	0.849	0.800	0.881	0.053
Avg/Model	0.952	0.934	0.902	0.875	0.844	0.818		
SD	0.029	0.030	0.038	0.051	0.032	0.021		

**Table 5:** Sensitivity performance of the CNN models across different MRI sequences.

Sensitivity	FLAIR	T1+C	ADC	T1	Diffusion	T2	Avg/Sequence	SD
VGG16	0.972	0.946	1.000	1.000	0.972	0.781	0.945	0.083
MobilenetV2	0.944	0.919	0.967	1.000	0.917	0.750	0.916	0.087
Resnet50	1.000	1.000	0.900	1.000	0.944	0.813	0.943	0.076
InceptionV3	0.972	0.865	0.967	1.000	0.889	0.781	0.912	0.083
Avg/Model	0.972	0.932	0.958	1.000	0.931	0.781		
SD	0.023	0.056	0.042	0	0.036	0.026		

**Table 6:** Specificity performance of the CNN models.

Specificity	FLAIR	T1+C	ADC	T1	Diffusion	T2	Avg/Sequence	SD
VGG16	1.000	1.000	0.688	0.600	0.471	0.913	0.779	0.224
MobilenetV2	0.889	0.950	0.938	0.750	0.647	0.870	0.841	0.118
Resnet50	0.926	0.850	0.875	0.600	0.765	0.870	0.814	0.117
InceptionV3	0.889	0.950	0.688	0.850	0.765	0.826	0.828	0.093
Avg/Model	0.926	0.938	0.797	0.700	0.662	0.870		
SD	0.052	0.063	0.129	0.122	0.139	0.035		

**Table 7:** Precision results.

Precision	FLAIR	T1+C	ADC	T1	Diffusion	T2	Avg/Sequence	SD
VGG16	1.000	1.000	0.860	0.780	0.800	0.930	0.895	0.097
MobilenetV2	0.920	0.970	0.970	0.850	0.850	0.890	0.908	0.055
Resnet50	0.950	0.930	0.930	0.780	0.890	0.900	0.897	0.061
InceptionV3	0.920	0.970	0.850	0.900	0.890	0.860	0.898	0.044
Avg/Model	0.948	0.968	0.903	0.828	0.858	0.895		
SD	0.038	0.029	0.057	0.059	0.043	0.029		

**Table 8:** AUC scores results.

AUC scores	FLAIR	T1+C	ADC	T1	Diffusion	T2	Avg/Sequence	SD
VGG16	1.000	0.991	0.887	0.875	0.755	0.867	0.896	0.091
MobilenetV2	0.965	0.978	0.983	0.925	0.853	0.852	0.926	0.060
Resnet50	0.965	0.957	0.844	0.818	0.907	0.894	0.898	0.059
InceptionV3	0.924	0.93	0.819	0.900	0.864	0.852	0.882	0.044
Avg/Model	0.964	0.964	0.883	0.880	0.845	0.866		
SD	0.031	0.027	0.072	0.046	0.064	0.020		

## Data Availability

All data generated or analyzed are available from the corresponding author upon reasonable request.
